# Screen-Printed Resistive Pressure Sensors Containing Graphene Nanoplatelets and Carbon Nanotubes

**DOI:** 10.3390/s140917304

**Published:** 2014-09-16

**Authors:** Daniel Janczak, Marcin Słoma, Grzegorz Wróblewski, Anna Młożniak, Małgorzata Jakubowska

**Affiliations:** 1 Institute of Metrology and Bioengineering, Faculty of Mechatronics, Warsaw University of Technology, A. Boboli 8 St., 02-525 Warsaw, Poland; E-Mails: marcin.sloma@mchtr.pw.edu.pl (M.S.); g.wroblewski@mchtr.pw.edu.pl (G.W.); maljakub@mchtr.pw.edu.pl (M.J.); 2 Institute of Electronic Materials Technology, Wolczynska 133 St., 01-919 Warsaw, Poland; E-Mail: amlozniak@poczta.onet.pl

**Keywords:** pressure sensors, graphene nanoplatelets, carbon nanotubes, printed electronics, screen printing, nanomaterials

## Abstract

Polymer composites with nanomaterials such as graphene nanoplatelets and carbon nanotubes are a new group of materials with high application possibilities in printed and flexible electronics. In this study such carbon nanomaterials were used as a conductive phase in polymer composites. Pastes with dispersed nanomaterials in PMMA and PVDF vehicles were screen printed on flexible substrates, and used as an active layer in pressure sensors, exploiting contact resistance phenomena. The relationship between resistance and pressure is nearly linear on a logarithmic scale for selected types of samples, and their response is several times higher than for similar sensors with graphite layers. The use of surfactants allowed us to fabricate evenly dispersed nanomaterials with different amount of nanoplatelets and nanotubes in the composites. The samples contained from 1.25 wt.% to 2 wt.% of graphene and 1 wt.% to 0.5 wt.% of nanotubes and exhibited diverse sheet resistivity. Experiments revealed the relationship between morphology and loading of functional phase in the polymer matrix and the sensors' sensitivity.

## 1. Introduction

Nowadays, a lot of attention is focused on the use of carbon nanostructures in various applications. Graphene and carbon nanotubes are attractive materials for reinforcing purposes and use as a functional phase in polymer composites, due to their excellent mechanical properties, with high thermal and electrical conductivity [1,2]. Such carbon nanomaterials are widely utilized in supercapacitors [3,4], FETs [5], transparent electrodes [6,7], and various chemical and biochemical sensors [8–11].

There are several ways to measure pressure changes with diverse sensor constructions, ranging from metal strain gauges, piezoresistive pressure sensors based on polycrystalline silicon through micromachined ceramic pressure sensor for high-temperature applications to highly sensitive flexible pressure sensors with microstructured rubber dielectric layers [12–15].

Some of the sensors operate on the basis of the contact resistance measurement. Shimojo *et al.* described a tactile sensor using conductive rubber with attached electrical wires [16,17]. The described sensor allows measurements in the range from 0 to 0.5 Mpa, with observed resistance changes from 1 kΩ to 100 Ω. For higher pressures measurements up to 2 MPa composite materials can be implemented, such as conductive composites filled with metal or carbon particles, or semi-conductive polymers [18,19].

Implementation of polymer composites containing carbon nanoparticles in flexible sensors which can be produced via printing techniques is also reported [20,21]. With the use of printing techniques sensors can adopt various shapes and sizes, extending their potential field of application [22,23]. In this paper, we report the fabrication of screen printed, resistive pressure sensors as a continuation of our first experiments concerning resistive layers made with carbon nanotubes [24]. Such sensors are alternatives to commonly used strain gauges based on tensometric bridges. Strain gauge bridges glued onto springy sensor structures undoubtedly are a disadvantage hindering their use in places with limited accessibility.

## 2. Experimental Section

### 2.1. Materials

**Figure 1.**
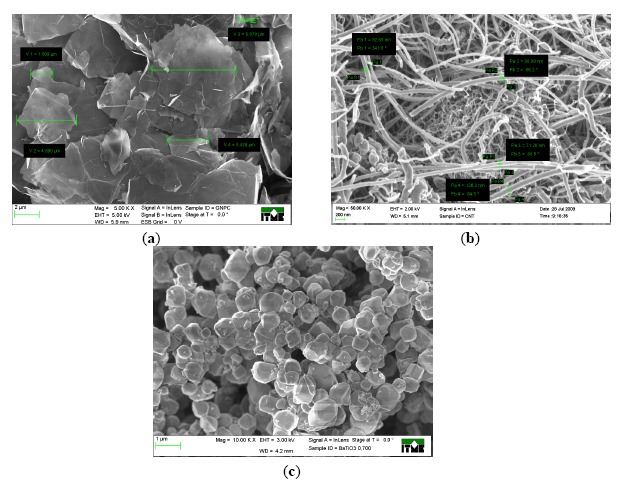
SEM image of (**a**) Graphene Nanoplatelets (GNP), (**b**) Multiwall Carbon Nanotubes (MWCNTs), (**c**) Barium titanate (BaTiO_3_) powder.

Graphene nanoplatelets were prepared from graphite using a modified Hummers method, and carbon nanotubes were synthesized by a catalyzed chemical vapor deposition method. Both materials were acquired commercially from Cheap Tubes Inc. (Grafton, VT, USA) Characteristic dimensions were estimated from scanning electron microscope (SEM) micrographs. Nanotube diameter was estimated in the range of 10–160 nm, and their length was between 0.5 and 5 μm (Figure 1a). Average thickness of graphene platelets was 10 nm and average particle diameter was 15 μm (Figure 1b). Additionally, barium titanate (BaTiO_3_) powder (particles size of 0.7 μm) from Inframat Advanced Materials (Manchester, CT, USA) was used as a filler to prepare dielectric pastes (Figure 1c).

Two types of polymer vehicles were selected to prepare carbon nanocomposites: a solution of M_w_ 350,000 polymethyl metacrylate (PMMA) in diethylene glycol butyl ether acetate (8 wt.%) and commercial vehicle (8155), a polyvinylidene fluoride (PVDF)-based polymer resin, acquired from Du Pont de Nemours (Wilmington, DE, USA).

### 2.2. Preparation

Compositions of graphene nanoplatelets and carbon nanotubes in PMMA and PVDF polymer vehicles were prepared by a modified mixing process used in thick film material preparation. PMMA solvent-based vehicle was produced by mixing polymer granulate with diethylene glycol butyl ether acetate solvent for 48 h with a magnetic blade mixer. The main purpose of the paste mixing process—to prepare a well-dispersed paste without agglomerates—was achieved by the sonication of carbon nanomaterials with dispersing agents in toluene for 60 min at room temperature. Uncontrolled, long time sonication process might influence negatively structure of materials and can reduce the diameter for the graphene flakes and shorten nanotubes length [25,26], therefore, the optimal time should not exceed two hours. Malialim AKM-0531 dispersing agent provided by NOF Corporation (Tokyo, Japan) was used for the surface treatment of carbon powders. It consists of two functional parts: a carboxylic acid anhydride group as a reactive part for interaction with the surface of the nanoparticles and the side chains that can react with polymer vehicle and thus improve dispersion. Addition of 5 wt.% of the dispersing agent with respect to the carbon fillers weight was sufficient to break agglomerates. After the partial evaporation of toluene, all samples were mixed with PMMA or PVDF vehicle in a mortar for 15 min. Afterwards pastes were rolled two times on the three-roll-mill with silicon carbide (SiC) rollers and 5 μm gap.

**Figure 2.**
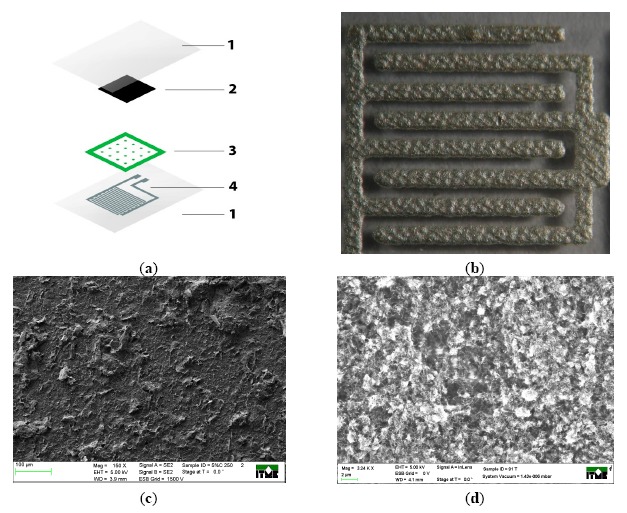
(**a**) Screen printed resistive pressure sensors: 1—polyester substrate, 2—pressure sensitive layers, 3—dielectric separator, 4—silver electrode. (**b**) comb electrodes, **(c–d)** SEM image of screen printed graphene (**c**) and carbon nanotube layer (**d**).

Sets of composite materials with different amounts of nanoplatelets and nanotubes were prepared. The loading of graphene nanoplatelets varied from 1.25 wt.% to 2 wt.% and the for carbon nanotubes it was 0.5 wt.% to 1 wt.%, respectively. Such polymer pastes were used for printing pressure-sensitive layers (Figure 2). The dielectric separator, presented as layer 3 in Figure 2, was printed with paste containing 76.3 wt.% of BaTiO_3_ powder in PVDF and PMMA vehicles.

Samples were printed with the screen printer AMI Presco 242 (Woodbridge, NJ, USA) with 200 mesh stainless steel screens for resistive pastes, and 68T polyester screens for conductive paths and dielectric layers. Afterwards, layers were cured in 120 °C for one hour, with exception to dielectric layers cured in 150 °C for half hour. Two comb electrodes were screen-printed on the bottom of polyester substrate foil (100 μm thickness) with silver paste L-121 from ITME (Warsaw, Poland).

Presented structures were deposited on 100 μm PET substrates, resulting in a total thickness of the sensors not exceeding 250 microns. The pressure-sensitive layer based on carbon nanocomposites resulted with thickness of about 10 μm. Dielectric separator resulted with a thickness of 20 μm, and silver electrodes 15 μm respectively. Substrates were preheated in 150 °C for one hour before printing to prevent thermal deformation during drying of the printed layers.

### 2.3. Measurement Procedures

Silver comb electrodes with 300 μm width paths and 350 μm spacing, cover a surface of 1.1 cm^2^ measurement area. All sensors have a dielectric separator made from barium titanate to ensure the isolation of the resistive layer from the comb electrodes in the unloaded position of the sensor. Sensor structures facing each other were placed in a hydraulic press. The pressure applied to the sensor varied from 10 N/cm^2^ to 15 kN/cm^2^. Contact resistance between sensor electrodes was measured as the response to the pressure applied to pressure-sensitive resistive area. For electrical measurements (resistance) a Keithley 2636A dual-channel source measure unit was used (Gertering, Germany).

### 2.4. Characterization

Thickness and profiles of the surface were measured with a Hommelwerke LV-50 contact profilometer (Villingen-Schwenningen, Germany). Observations were done on a Carl Zeiss Stemi 2000-C optical microscope (Oberkochen, Germany), and a Carl Zeiss AURIGA CrossBeam Workstation scanning electron microscope.

## 3. Results and Discussion

Resistance measurements of screen printed layers showed that the increase of the carbon filler loading in the polymer composite causes the decrease of their sheet resistivity. Moreover, the employment of PMMA in the vehicle resulted in the lower sheet resistivity than for PVDF vehicle. Surface resistance measurements for all of the composites are presented in Table 1.

Mechanical fatigue tests after 50,000 bending cycles confirmed good adhesion to the substrate and showed slight changes in the sheet resistivity of the samples. The change observed was less than 5% for all samples with both types of resin.

The conductivity of composites depends on many factors, including type of resin, type of the filler and loading. Carbon nanotubes and graphene nanoplatelets are materials with very different aspect ratios. This is the reason the properties of their composites differ significantly from each other. Filler loadings allowing them to reach the percolation threshold, at which the printed layer begins to conduct electricity, are different for both types of nanoparticles. For graphene nanoplatelets dispersed in PMMA matrix it is about 1.25 wt.%, while for PVDF matrix it is 1.5 wt.%, respectively. For MWCNT this value is appropriately 0.1 wt.% in PMMA and 0.5 wt.% in PVDF, respectively. All values are denoted for the loading of functional phase in the paste. Electrical properties of carbon composites vary for both fillers with the same content. Therefore, direct comparison of the layers with the same loading of functional filler is not possible. The authors decided to compare layers exhibiting the same sheet resistivity. Pastes were selected in order to obtain layers with high sheet resistivity promoting high sensitivity of printed sensors.

**Figure 3.**
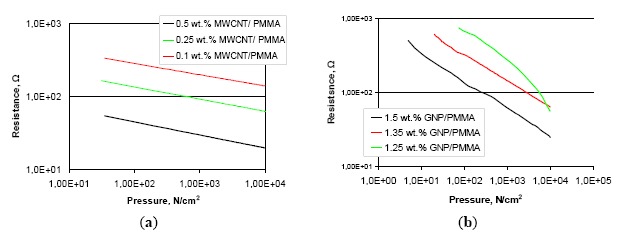
Characteristics of the pressure sensors based on the PMMA resin pastes with: (**a**) multiwalled carbon nanotubes, (**b**) graphene nanoplatelets.

Resistance change responses under applied pressure for sensors with PMMA composite layers, are presented in the Figure 3. Layers made with carbon nanotubes are shown in Figure 3a and for graphene nanoplatelets in Figure 3b, respectively.

Measurement resolution of the sensor is directly related to the sheet resistivity of active composite layer, for both types of nanomaterials used. During pressure application, the resistance of the sensors changed by up to 700 Ω, measured at the contact pads of silver electrodes. The lower resistance value of the sensor compared with the surface resistivity of the carbon layers is the result of low resistance measured through a thin (10 μm) active composite layer. We observed also that sensors with graphene nanoplatelets exhibited a higher resolution compared with the corresponding sensors with carbon nanotubes. This is related to the higher surface area of graphene nanoparticles. Interestingly, we observed that relationship between resistance and applied pressure is linear on a double logarithmic scale. This is a useful result for accurate measurements in the realization of pressure sensors. A linear relation was observed for sensors with layer loadings above the percolation threshold. Layers on the edge of the percolation threshold, though resulting in higher sensitivity sensors, exhibited nonlinear characteristics.

**Figure 4.**
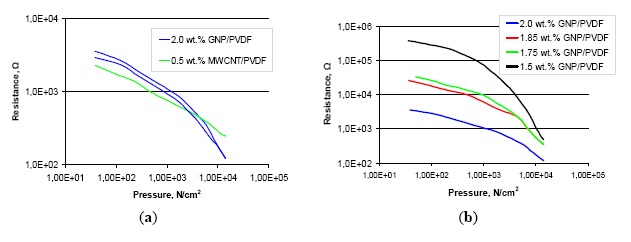
Characteristics of the pressure sensors made with the PVDF resin pastes: (**a**) MWCNT and GNP pressure-sensitive layers with 150 kΩ/sq sheet resistivity, (**b**) GNP pressure-sensitive layer with sheet resistances from 150 kΩ/sq to 5 MΩ/sq.

Figure 4 shows the characteristics obtained for sensors with sheet resistivity from 150 kΩ/sq to 5 MΩ/sq. Much larger resistance changes were observed for sensors made of PVDF pastes that for those made of PMMA pastes. An increase of sensitivity was observed for sensors with sheet resistivity above 2.5 MΩ/sq. For sensors with comparable sheet resistivity (1 MΩ/sq), the PVDF layer allowed us to obtain significantly higher sensor sensitivity. We observed a small hysteresis for sensors containing graphene nanoplatelets as shown in Figure 4a. However, sensors with carbon nanotubes, despite negligible hysteresis, give less repetitive measurement results in the following measurement cycles. This is related to the change in layer structure in which deformed nanotubes create additional contacts between each other, while the planar arrangement of platelets preserves them in the primary location.

## 4. Conclusions

The presented results show that the printed pressure sensor resolution depends on the filler material, filler loading and resin type. Sensors with a pressure sensitive layer made with graphene flakes have a larger contact surface than similar layers made with carbon nanotubes, what causes significant improvement of the sensors' resolution. Results of mechanical fatigue tests proved the high durability of the sensors with almost unchanged resistance of the layers.

Structures based on PMMA resin for both carbon fillers display linear characteristics on a logarithmic scale. The sensitivity of the sensor increases with increasing sheet resistance of the measuring layer. For the best CNT sensors with 0.1 wt.% filler content the resistance changed from 360 Ω to 140 Ω in the measuring range. For sensors with 1.25 wt.% GNP content resistance changes were twice as large, from 750 Ω to 60 Ω but the sensor characteristics are no longer linear. Sensors based on PVDF composites were characterized by a much higher sensitivity compared to previous ones, and for sensors with graphene filler linear characteristics were not observed, and it is a big disadvantage. In the future composites with higher filler content need to be checked. For sensors with 1.5 wt.% GNP content in PVDF resin resistance changes from 380 kΩ to 490 Ω were observed.

## Figures and Tables

**Table 1.** Results of the surface resistivity measurements.

**No.**	**Composite (Filler/Matrix)**	**Surface Resistivity, kΩ/sq**
1	0.5 wt.% MWCNT/PMMA	39 ± 3
2	0.25 wt.% MWCNT/PMMA	142 ± 14
3	0.1 wt.% MWCNT/PMMA	798 ± 56
4	1.5 wt.% GNP/PMMA	603 ± 44
5	1.35 wt.% GNP/PMMA	898 ± 63
6	1.25 wt.% GNP/PMMA	1007 ± 81
7	0.5 wt.% MWCNTGNP/PVDF	147 ± 11
8	2 wt.% GNP/PVDF	154 ± 16
9	1.85 wt.% GNP/PVDF	1012 ± 79
10	1.75 wt.% GNP/PVDF	2482 ± 177
11	1.5 wt.% GNP/PVDF	4992 ± 353
